# The Role of Wearable Devices in Chronic Disease Monitoring and Patient Care: A Comprehensive Review

**DOI:** 10.7759/cureus.68921

**Published:** 2024-09-08

**Authors:** Eman A Jafleh, Fatima A Alnaqbi, Hind A Almaeeni, Shooq Faqeeh, Moza A Alzaabi, Khaled Al Zaman

**Affiliations:** 1 College of Dentistry, University of Sharjah, Sharjah, ARE; 2 College of Medicine, University of Sharjah, Sharjah, ARE; 3 Internal Medicine, Cleveland Clinic Abu Dhabi, Abu Dhabi, ARE; 4 General Medicine, Cleveland Clinic Abu Dhabi, Abu Dhabi, ARE

**Keywords:** chronic diseases, patient-centered care, patient monitoring, patient outcome, public health, telemedicine, wearable devices

## Abstract

Wearable health devices are becoming vital in chronic disease management because they offer real-time monitoring and personalized care. This review explores their effectiveness and challenges across medical fields, including cardiology, respiratory health, neurology, endocrinology, orthopedics, oncology, and mental health. A thorough literature search identified studies focusing on wearable devices’ impact on patient outcomes. In cardiology, wearables have proven effective for monitoring hypertension, detecting arrhythmias, and aiding cardiac rehabilitation. In respiratory health, these devices enhance asthma management and continuous monitoring of critical parameters. Neurological applications include seizure detection and Parkinson’s disease management, with wearables showing promising results in improving patient outcomes. In endocrinology, wearable technology advances thyroid dysfunction monitoring, fertility tracking, and diabetes management. Orthopedic applications include improved postsurgical recovery and rehabilitation, while wearables help in early complication detection in oncology. Mental health benefits include anxiety detection, post-traumatic stress disorder management, and stress reduction through wearable biofeedback. In conclusion, wearable health devices offer transformative potential for managing chronic illnesses by enhancing real-time monitoring and patient engagement. Despite significant improvements in adherence and outcomes, challenges with data accuracy and privacy persist. However, with ongoing innovation and collaboration, we can all be part of the solution to maximize the benefits of wearable technologies in healthcare.

## Introduction and background

The integration of wearable health devices in healthcare systems has transformed the management of chronic conditions. These tools, from wrist sensors to implanted monitors, provide continuous monitoring and personalized interventions.

Wearable health devices have demonstrated significant potential in effectively managing chronic diseases. For instance, continuous glucose monitors (CGMs) have transformed diabetes management by providing real-time glucose readings, leading to more precise insulin dosing and improved glycemic control. Studies have shown that CGMs can substantially reduce hemoglobin A1c levels in type 1 diabetes, enhancing overall diabetes management [1]. Similarly, wearable devices that monitor heart rate (HR), blood pressure (BP), and pulse during moderate exercise can aid in managing cardiovascular diseases by identifying arrhythmias, monitoring BP, and promoting critical cardiovascular function through exercise [2].

Despite the potential benefits, several challenges impede the widespread clinical use of wearable health devices. The accuracy and reliability of the data collection are major concerns. Although technological advances have improved the accuracy of wearable sensors, differences in data accuracy, especially under physical conditions, are a significant issue [3]. Furthermore, integrating wearable device information with existing health records creates operational challenges. Standardized protocols and robust data structures are needed to ensure flow and usability [4]. Data privacy and security are major concerns for wearable healthcare devices. The ongoing collection and transmission of sensitive health information exposes users to potential data breaches and unauthorized access. Ensuring the confidentiality and accuracy of health information requires robust security measures, including encryption, secure data storage, and compliance with regulatory standards such as the Health Insurance Portability and Accountability Act (HIPAA) in the United States and compliance with the General Data Protection Regulation (GDPR) in Europe [5]. Addressing these issues is not just important; it is urgent to build trust among users and healthcare providers and facilitate the widespread adoption of wearable healthcare technologies.

Furthermore, the cost of wearable health devices and accessories can hinder widespread adoption, especially in resource-constrained settings. Although the price of these devices has declined over time, money and initial incorporation into hardware, software, and training may be relevant to patients and healthcare professionals in reducing wear and preventing disease complications [6]. In addition to technical and financial challenges, user adherence and engagement are important factors affecting the effectiveness of wearable health devices. The success of these devices depends mainly on the willingness of patients to continue to use them and to engage with the information they provide. Factors such as device comfort, ease of use, and perceived usefulness of the information provided play an essential role in user retention. Studies have shown that personalized information and actionable insights derived from wearable device data can increase user engagement and lead to better health [3].

The potential of wearable health devices extends beyond individual illness prevention to broader public health interventions. Aggregate data from wearable devices can offer valuable insights into public health issues, disease outbreaks, and the effectiveness of public health interventions. This potential for broader impact is a reason for optimism, as wearable devices can aid in conducting extensive epidemiological studies using continuous data on physical activity, sleep patterns, and other health metrics in diverse populations [5]. This information can be instrumental in shaping public health policies and programs to enhance the population’s health.

In summary, wearable health devices have great potential for improving the monitoring and management of chronic diseases by providing real-time insights and personalized interventions for patients. However, addressing challenges related to data accuracy, privacy, cost, and user engagement is crucial to fully achieve this potential. If these obstacles can be overcome, wearable health devices can become a cornerstone of modern healthcare, contributing to improved healthcare quality and broader public health coverage.

## Review

Methodology

Research Question and Search Strategy

The research question proposed in our review article is: How effective are wearable health devices in monitoring chronic diseases, and how do they improve patient outcomes and prognosis?

A thorough literature search was conducted using the Scopus database. The data extraction on the Scopus database was conducted on July 11, 2024. The search queries were keyword combinations including “wearable,” “device,” “disease,” and “monitor” to determine relevant research articles in the title, abstract, and keywords. Moreover, the terms “cardio,” “endocrin,” “respiratory,” “neurolog,” “cancer,” “ortho,” and “mental” were selected for inclusion in the research for analysis based on the information identified in the title fields of articles in the database.

Search Query

TITLE-ABS-KEY ( wearable AND device ) AND TITLE-ABS-KEY ( "disease" OR "monitor*" ) AND TITLE ( "cardio*" OR "endocrin*" OR "respiratory" OR "neurolog*" OR "cancer" OR "ortho*" OR "mental*" ) AND ( LIMIT-TO ( SUBJAREA, "MEDI" ) ) AND ( LIMIT-TO ( DOCTYPE, "ar" ) OR LIMIT-TO ( DOCTYPE, "re" ) ) AND ( LIMIT-TO ( LANGUAGE, "English" ) ) AND ( LIMIT-TO ( SRCTYPE, "j" ) ) AND ( LIMIT-TO ( PUBSTAGE, "final" ) )

Study Selection

The studies were carefully selected based on specific criteria to gain insights into the impact of wearable health devices on patient outcomes. We included articles identified and retrieved from the databases, focusing on wearable health devices in various medical fields, such as cardiology, respiratory health, neurology, endocrinology, orthopedics, cancer, and mental health. These studies needed to assess the impact of wearable devices on patient outcomes, such as disease management, quality of life (QOL), and clinical results, and address challenges related to the implementation, adoption, and usability of these devices. The study only included the articles if they had been published in a journal, excluding some sources, such as books, preprints, conference abstracts, and reports. The selected studies needed to assess the impact of wearable devices on patient outcomes such as disease management, QOL, clinical results, and patient adherence. Only peer-reviewed English-language papers were considered, focusing on clinical trials, cohort studies, case-control studies, and systematic reviews that provided empirical data or comprehensive analysis. Studies that did not directly assess patient outcomes or focused on unrelated technological aspects were excluded.

**Figure 1:**
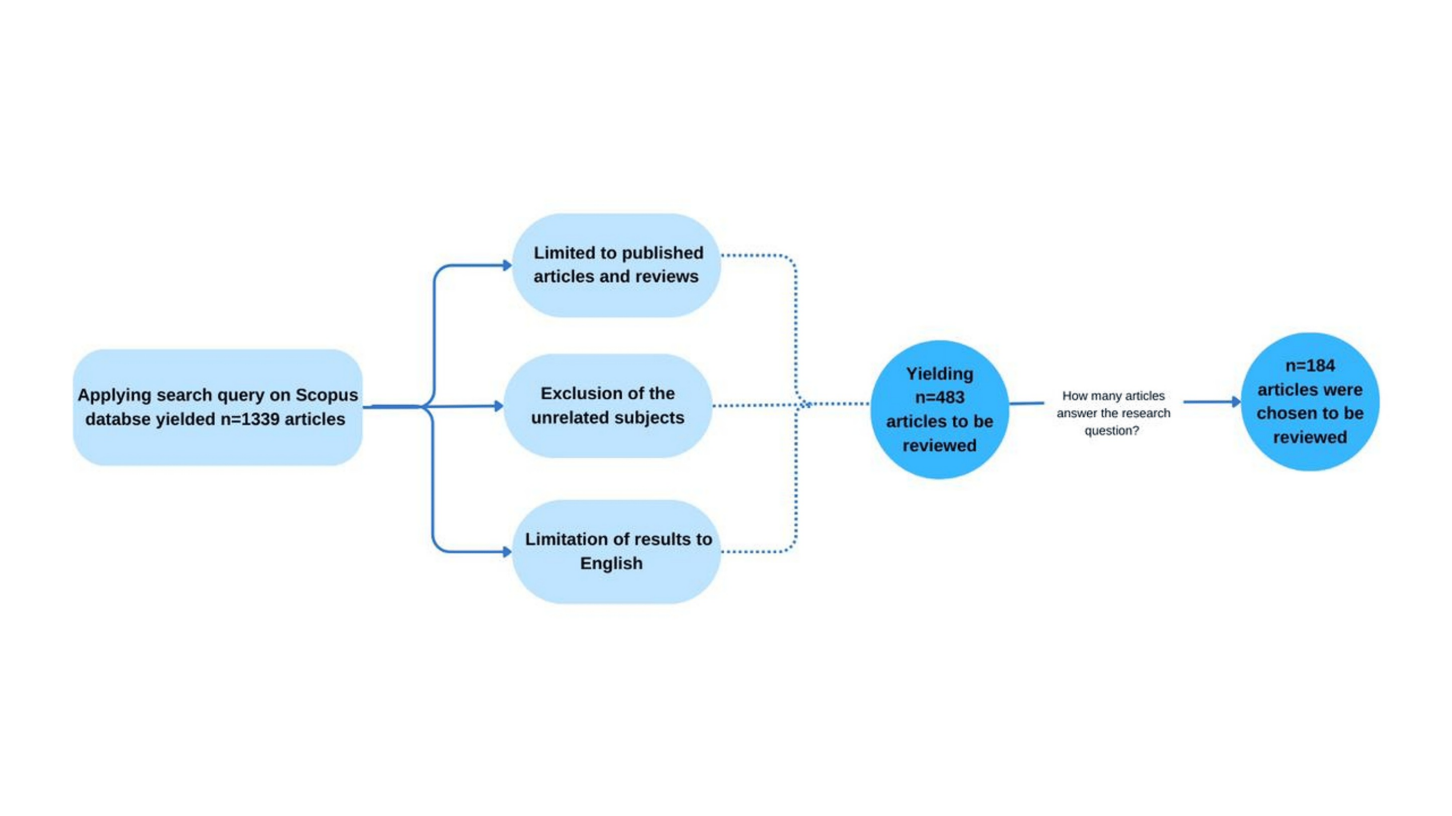
Flowchart of the literature search and selection process

An initial search on the Scopus database identified 483 studies. The authors evaluated the studies for relevancy and applicability to the research question. This resulted in the exclusion of 299 studies, leaving 184 articles to be selected for the review article. Figure 1 shows a flowchart of the literature search and selection process.

Data Charting and Analysis

A narrative synthesis technique was used to evaluate the data following the classification of the data according to the several health domains (cardiology, respiratory health, neurology, endocrinology, orthopedics, cancer, and mental health). The usage of wearable technology in various domains, its influence on patient outcomes, and the difficulties in utilizing it were taken into consideration while developing the themes. In these subsections, the results were presented methodically, emphasizing significant findings and pointing out gaps in the literature.

Role of wearable technologies in cardiology

Monitoring Hypertension

Wearable and smartphone technologies have become essential tools in managing and preventing hypertension. Ambulatory BP (ABP) and home BP (HBP) monitoring are recommended for hypertension management. However, traditional HBP protocols may not capture diurnal fluctuations, potentially linked to adverse cardiovascular outcomes. In an observational study, 62 treated hypertensive patients underwent 24-hour ABP measurements using an oscillometric upper-arm monitor and three sessions of seven-day/six-time-daily wearable HBP measurements with the HeartGuide, a wrist-worn oscillometric BP monitor. The wearable HBP measurements demonstrated substantial reliability (intraclass correlation coefficient (ICC) 0.883-0.911) and good reproducibility (Cohen’s kappa = 0.600). Wearable HBP monitoring following a structured daily schedule shows reliable and reproducible results. Patients with uncontrolled wearable HBP may benefit from repeated HBP or ABP measurements for risk assessment [7]. Recent guidelines advocate for increased use of out-of-office BP measurements to aid hypertension management [8-10]. ABP monitoring (ABPM) and HBP monitoring are accepted methods for out-of-office BP measurement. However, these methods have limitations regarding the number of measurements they can provide. The HeartGuide, a wrist-type BP monitoring device, employs oscillometric measurement directly to assess BP values. Equipped with a position sensor, it ensures correct wrist positioning relative to heart level during measurement. A study comparing the HeartGuide with traditional ABPM in office and out-of-office settings among 50 adult outpatients demonstrated that differences within ±10 mm Hg were observed in 58.7% and 47.2% of measurements in office and out-of-office settings, respectively. Statistical analysis showed no significant temporal trend in the differences between out-of-office BPs measured by the two devices. In conclusion, the differences observed between wearable BP monitoring and ABPM were acceptable in and out of clinical settings [11,12].

Furthermore, a comparative study evaluated the HeartGuide and another wearable watch-type BP monitor (Smart Wear) against conventional ambulatory BP monitors and auscultatory sphygmomanometry. Among 159 patients, HeartGuide provided comparable BP values to standard devices for single- and long-term measurements. In contrast, the infrared smart device failed to consistently acquire valid measurement data, showing poor agreement (ICC <0.4) with conventional methods [13].

These studies underscore the potential of wearable BP monitoring devices like the HeartGuide to enhance hypertension management by offering reliable, continuous BP monitoring both within and outside clinical settings.

Detecting and Monitoring Arrhythmias

Wearable devices equipped with optical sensors, such as smartwatches, are widely used to measure user pulse rates. Algorithms have been developed to leverage pulse wave data from these devices to detect atrial fibrillation (AF) and atrial flutter [14,15]. For example, an application on the Apple Watch uses intermittently detected pulse rate data in an algorithm designed to identify episodes suggestive of AF. The effectiveness of these algorithms was demonstrated in the Apple Heart Study, a prospective single-arm study aimed at evaluating a smartwatch-based irregular pulse notification algorithm’s ability to identify AF. Among participants who received notifications of an irregular pulse, subsequent ECG patch readings confirmed AF in 34%, with 84% of notifications being concordant with AF [16].

Similarly, the Fitbit Heart Study, a single-arm remote clinical trial, evaluated the validity of a novel photoplethysmography (PPG)-based software algorithm for detecting AF. This proprietary algorithm analyzes pulse waveform intervals during stationary periods for participants wearing compatible wrist-worn trackers or smartwatches. Enrollment reached 455,699 participants. Those detected with an irregular heart rhythm underwent telehealth visits and were subsequently monitored with a one-week single lead ECG patch, demonstrating an irregular heart rhythm detection positive predictive value (PPV) of 98.2% (95% CI, 95.5-99.5%). Participants aged ≥65 years showed a PPV of 97.0% (95% CI, 91.4-99.4%) [17]; in another approach, a study evaluated a ring-type wearable device called CardioTracker (CART) utilizing deep learning analysis of PPG signals for AF detection. Conducted with patients undergoing cardioversion for persistent AF, this study compared PPG signals with simultaneous single-lead electrocardiography. The CART device achieved high diagnostic accuracy, sensitivity, specificity, PPV, and negative predictive value (NPV) of 96.9%, 99.0%, 94.3%, 95.6%, and 98.7%, respectively [18].

Moreover, a patch-type device, the AT-Patch, was assessed in a study involving adults at high risk of new-onset AF. Attached for 11 days, the AT-Patch recorded continuous heart electrical activity and detected AF in 3.4% of participants, as confirmed by two independent cardiologists. The device’s reliability was supported by an overall Cohen κ coefficient of 0.840 [19]. In comparison, the Zio® Patch by iRhythm Technologies is a single-use, waterproof, continuous ECG monitor worn for up to 14 days. In a trial comparing it to conventional 24-hour Holter monitoring, the Zio Patch detected 57% more significant clinical events (96 vs. 61 events, p < 0.001). Participant preference favored the Zio Patch due to its comfort and minimal disruption to daily activities [20]. Additionally, a multicenter randomized clinical trial investigated AF screening in older individuals with hypertension using continuous electrocardiographic (cECG) patch monitors and HBP machines. Twice-daily AF screening with the home BP monitor during cECG monitoring periods achieved a sensitivity of 35.0%, specificity of 81.0%, PPV of 8.9%, and NPV of 95.9% [21].

These studies collectively underscore the significant advancements and potential of wearable devices with optical sensors in detecting and managing AF. They offer noninvasive, continuous monitoring capabilities that enhance clinical outcomes and patient care.

Cardiac Rehabilitation (CR)

CR is a comprehensive, evidence-based program integrating exercise training, health education, physical activity promotion, and counseling to manage cardiovascular risk factors effectively. Wearable devices play a crucial role in this context by monitoring physiological functions such as HR, movement, sleep, ECG analysis, and more, providing valuable biometric data [22,23]. Despite barriers like inconvenience and cost associated with in-person sessions, wearable devices have the potential to enhance accessibility and affordability, facilitating reliable care from the comfort of patients’ homes [24,25]. CR’s most commonly used wearable devices include HR monitors, ECG monitors, accelerometers, pedometers, and health watches [26-29]. These devices support various aspects of CR, offering real-time data that aids in monitoring and managing patient progress remotely. Home-based CR (HBCR) emerges as a promising alternative to center-based programs, supported by evidence showing significant improvements in cardiovascular health metrics when using wearable ECG or HR monitoring devices. For instance, a meta-analysis encompassing 15 randomized controlled trials (RCTs) with 1,314 participants highlighted the substantial benefits of HBCR using wearable technology. Significant improvements were observed in left ventricular ejection fraction (MD = 2.12, 95% CI 1.21, 3.04), six-minute walk distance (MD = 40.00 meters, 95% CI 21.72, 58.29), and peak oxygen intake (MD = 2.24 ml/(min kg), 95% CI 1.38, 3.10) compared to traditional center-based rehabilitation [30]. In another study focused on patients post-ablation for AF, tele-monitored CR demonstrated effectiveness in improving cardiovascular fitness. Participants in the intervention group, guided by a mobile application, showed significant increases in mean peak oxygen consumption (VO2peak) from baseline to 12 weeks (intervention group: 27.3 ± 5.6 ml/(min kg); control group: 22.9 ± 6.3 ml/(min kg), both p < 0.01), underscoring the potential of telehealth approaches in enhancing rehabilitation outcomes [31]. Additionally, a prospective RCT investigated the impact of home-based telemonitored Nordic walking (NW) in heart failure patients, including those with cardiac implantable electronic devices. NW resulted in significant improvements in VO2peak (NW group: 18.4 ± 4.1 ml/kg/min vs. control group: 16.1 ± 4.0 ml/kg/min, p = 0.0001), exercise duration, six-minute walking test distance, and QOL compared to usual care alone [32].

One of the primary obstacles to participating in CR is the distance patients must travel to the rehabilitation center. A RCT conducted in Greece aimed to compare the effectiveness of a real-time online supervised CR exercise program using wearable technology versus a traditional gym-based rehabilitation program. Over 24 weeks, the online group exhibited significant improvements in mean daily steps (p < 0.05) and mean daily distance (p < 0.05) compared to the gym-based group. Both groups showed statistically significant improvements in all assessed variables at 24 weeks based on paired-sample t-tests (p < 0.001). Positive correlations were found between VO2peak and mean daily distance (r = 0.375), and negative correlations between VO2peak and muscle mass (r = -0.523) and fat mass (r = -0.460) [33].

Wearable devices have significantly improved cardiorespiratory fitness, QOL, and physical activity levels in CR settings. They offer a promising avenue for long-term engagement in physical activity post-CR, highlighting their potential as valuable components or alternatives to traditional center-based rehabilitation programs.

Role of wearable technologies in respiratory health

Wearable devices are transforming respiratory health management by continuously monitoring respiratory rate, oxygen saturation, and other vital indicators. Real-time data from these devices helps in the early diagnosis and treatment of a number of respiratory diseases, such as asthma and chronic obstructive pulmonary disease (COPD). Bentley et al. explored the feasibility and acceptability of the Chronic Obstructive Pulmonary Disease Self-Management Activation Research Trial (COPD-SMART) mHealth intervention, which involved using the Fitbit activity tracker and smartphone application to assist COPD patients to complete or enhance physical activity after pulmonary rehabilitation (PR). Although many participants dropped out due to ill health, technology burden, or technical issues, those who completed the study demonstrated enhanced physical activity and exercise capacity. Motivational feedback and physical activity monitoring were achieved with the aid of Fitbit trackers. The results show that with the aid of wearable devices, mobile health (mHealth) interventions can improve the self-management of COPD. The study recommended that the devices be more appealing and less complicated to increase compliance and lower attrition [34].

Moreover, Vooijs et al. examined the validity and usability of cheap gadgets with internet-connected accelerometers, such as Fitbit Ultra and personal activity monitor, to monitor physical activity in COPD patients. It was noted that both devices have acceptable validity and usability, and patients stated their willingness to use such devices in the future. However, the patient’s feedback revealed issues such as discomfort and problems with data synchronization. In light of the findings, inexpensive accelerometers can contribute to the objective assessment of physical activity and self-management in COPD [35].

Furthermore, Castner et al. assessed the usability and feasibility of using Fitbit Charge™ against the Actigraph wGT3+ device for sleep time analysis in women with uncontrolled asthma. In the study, a high sensitivity (97%) of Fitbit was observed for the detection of sleep. At the same time, sleep efficiency was overestimated compared to that of actigraphy. However, fitness trackers such as Fitbit Charge™ are practical and efficient in assessing sleep disorders in asthma patients. Notably, one of the issues observed was that Fitbit often overstated the sleep parameters [36]. In addition, Bian et al. examined the correlation between QOL with asthma and sleep quality and physical activity generated by Fitbit in adolescents with asthma. Participants wore Fitbit Charge HR wristbands to collect real-time data on their physical activity and sleeping patterns. The findings of this study indicate a weak but statistically significant negative correlation between Fitbit sleep quality and pediatric asthma impact and a moderate correlation (average r = -0.31, p = 0.01) between Fitbit sleep qualities and sleep disturbance measures in children with asthma. Based on the findings, wearable technology should be incorporated into personalized asthma self-management among adolescents. The limitation of the study was the small sample size, which lacked enough evidence to draw a conclusion [37]. Another study by Varas et al. investigated how community-based PR, which incorporated a wearable pedometer as an intervention, impacted the physical activity levels of patients with COPD. As for the study findings, the pedometer-based experimental group had improved exercise capacity, increased activity levels, and enhanced QOL compared to the control group. The study also showed valuable information about the positive impacts of wearable devices in modifying the long-term effect of PR programs and interventions on the health of COPD patients. However, there were complaints and concerns regarding the participant's inconsistent motivation and adherence to the program [38].

In addition, Kouis et al. examined the impact of wearable technology on the behavioral responses of children with asthma to the extent that they follow public health guidelines during desert dust storms (DDS) in Cyprus and Crete. In school, the children aged six to 11 years were permitted to wear smartwatches with pedometers and GPS to collect data in real conditions. Compared to the control group, the intervention group, which received timely alerts and recommendations from DDS, reduced their time spent outside by 62.4 minutes and steps by 1,039. It also showed that health behaviors could be periodically monitored and altered with wearables to help reduce the impact of asthma and support the overall well-being of children during DDS events. However, researchers noted children's self-directedness towards the device despite the helpfulness of the planning tool; collecting data for the study was difficult, especially when children were at their most active best after school time [39]. In another study, Kim et al. discussed the possibility of integrating the MyHealthKeeper app with Samsung Health and electronic health record systems with Samsung Charm Wearable. It was applied in patients with obstructive sleep apnea to assess the effects of health outcomes and response. The Samsung Charm is worn at the wrist of the patient, tracks the patient’s steps, and relays them to the application. The outcomes showed a significant reduction in weight in both the app-plus wearable and app-only groups (average: 1.4 kg and 2 kg; 95% CI: 0.9-1.9; p < 0.001), with higher daily steps recorded in the app-plus-wearable group (8,165 steps) compared to the app-only group (6,034 steps). Introducing wearable devices increased patient engagement and self-management of obesity-related respiratory diseases. However, the challenges were the high incidence of nonadherence and continuous follow-ups [40].

Kirszenblat and Edouard confirmed the reproducibility and reliability of Withings ScanWatch for measuring oxygen saturation (SpO2) levels. Participants placed two ScanWatch devices on their wrists to measure SpO2, which was then synced to the Health Mate application through Bluetooth. The study showed the efficacy and safety of the device in the management of such conditions as COPD and sleep apnea. The user-friendly design indicates a high adherence rate and can be used repeatedly to enhance disease management since the condition will be closely monitored [41]. Also, Orme et al. examined the acceptability of a waist-worn inclinometer integrated with an application for reducing sedentary behavior among COPD patients. Feedback showed sitting time, stand-ups, and steps taken with vibration, motivating the user to get up and move after sitting for extended periods. The study was also proven to be feasible. However, ways to enhance retention and adherence need to be done. Based on the results and limitations of the study, the intervention demonstrated a possibility of enhancing activity levels and PR [42].

Wu et al. explored the effect of wearable technology on COPD management among patients. The participants used devices to help monitor the extent of oxygen saturation, pulse rate, and activity level. For some, the possibility of maintaining daily life and controlling exacerbation was actual and realistic. However, they point out possible issues concerning usability, independence, and the data itself [43]. Furthermore, Moraveji et al. showed how health tags worn on the undergarments of patients can be helpful in COPD management by offering constant respiratory data. These devices showed high adherence, and 78.7% of each 24-hour cycle was worn. They also reported high data quality and coherence throughout multiple types of activity and assumed potential for continuous monitoring. Some wearability concerns were identified, like adhesion damages to clothing, but Health Tags provided useful longitudinal information about disease severity and possible worsening [44].

In a systematic review, Buekers et al. selected and reviewed 71 studies to evaluate the use of wearable pulse oximeters in telemonitoring COPD patients. For daily SpO2 spot check purposes, these devices demonstrated great potential for long-term use in chronic condition monitoring. The SpO2 values were vital in initiating medical alarms and establishing prognostic models for worsening. However, due to the absence of firmly established procedures for their implementation, it was not easy to draw definite conclusions on their efficiency. The review also highlighted the need to implement values for individual risk thresholds and predictive algorithms to enhance telemonitoring [45].

Moreover, Kwon et al. also introduced and evaluated an mHealth system that included self-monitoring wearable pulse oximeters connected to COPD rehabilitation applications on mobile devices. Patients continuously used pulse oximeters to monitor oxygen saturation during the 12-week exercise program. The use of wearable technology allowed for real-time tracking of the oxygen levels and activity, which motivated the patient and ensured compliance with the treatment plan. The results show enhanced COPD assessment test scores, observed by the intervention period’s end. These results suggest utilizing wearable technology and mobile apps for chronic diseases such as COPD [46].

Furthermore, Buekers et al. evaluated the possibility of using WristOx2 pulse oximeters for SpO2 monitoring in patients with COPD. They wore the device on the wrist and obtained quality data about oxygen saturation when patients were going through home routines. The findings also revealed fluctuations that spot checks might overlook. Notably, the patients evidenced high compliance, specifically during rest periods. The study noted the effectiveness of continuous SpO2 monitoring for enhancing the management of COPD patients, as it illustrated that detailed data acquired in real-time could significantly facilitate interventions. However, motion artifacts reduced data quality during moderate-to-vigorous physical activity [47].

Waschki et al. assessed the prognostic value of physical activity in COPD patients through the Sense-Wear armband, which monitored activity and steps in the patients. The armband, worn around the arm, gave real-time readings over five to six days. The researchers found that the intensity of physical activity was the most significant predictor of all-cause mortality, even better than the traditional risk factors. The outcomes supported the effectiveness of wearable devices in assessing and increasing the prognosis and care of COPD patients [48]. In another study, Vlamos evaluated the feasibility of a home telemonitoring system for idiopathic pulmonary fibrosis (IPF) patients using Bluetooth medic alert pulse oximeters and BP meters. The patients wore these to constantly track vital signs, sending information wirelessly to the healthcare personnel. The researcher found high compliance and acceptability of the system, and more importantly, the system facilitated timely medical interventions. The continuous monitoring features of wearables show their application in managing chronic conditions like IPF [49].

In addition, Liu et al. developed an algorithm for respiratory rate monitoring based on the data collected by a wearable sensor that measures three-dimensional (3D) acceleration. It uses adaptable band-pass filtering and principal component analysis to process the signals. The researchers found that the monitoring accuracy was relatively higher around the proposed method, with a mean absolute error of around 10%, compared to various methods. Real-time respiratory rate assessment was achieved in various activities and during overnight sleep using the wearable device, which is essential for continuous health assessment and early identification of respiratory problems. However, challenges included motion artifacts and data integration issues, which were solved through proper algorithms and advanced media access control [50].

Similarly, Singh et al. also designed and assessed the potential of a wearable platform to measure the respiratory rate with a higher reliability level based on adaptive optical methods. Using the wearable device equipped with a vertical cavity surface emitting laser-based optical diffuse reflectance technique, the wearable technology provided good agreement with manual counts, with deviations within 2 bpm. The outcomes showed that consistent, real-time assessment of respiratory rates could improve the ways of identifying respiratory distress and minimizing manual errors. The primary concerns included data quality issues when patients are transferred from one location to another and the difficulty of making users adhere to the set processes. However, these challenges do not negate the application of wearable devices in clinical settings for respiratory monitoring, especially in chronic conditions at home [51].

Role of wearable technologies in neurology

Seizures

Specific seizure types can be detected using wearable devices, which are available in abundance and are being approved for the management of epilepsy. They are increasingly used to detect, track, and manage seizures [52-56]. These devices include surface electromyographic signals, electrodermal activity, 3D accelerometry, and HR variability that measure features of seizures, which are either autonomic or motor [57]. Generally, responses from patients with epilepsy on wearable devices were positive, with a preference for wristbands [52]. A wrist-worn accelerometer device with a built-in microelectromechanical system can be used to distinguish between convulsive psychogenic non-epileptic seizures (ESs) and convulsive ESs by measuring Poincaré-derived temporal variations (sensitivity: 70.27%; specificity: 86.84%. The data of the movement is recorded and saved and can be retrieved from a flash drive. Although this approach is considered low-cost, accurate, and effective for long-term usage, a drawback is the required trained personnel to confirm the diagnosis, as seizure events and manifestations vary between individuals [58]. A study in patients with epilepsy using one or multiple devices simultaneously reported the best results about both usage and manipulation. The Biovotion arm-worn device had 94% acceptable quality, and signal quality indices (SQI) were 90%, showing high agreement with human labels. The GENEActiv device was documented as providing the best results concerning comfort during both sleep and long-term comfort. However, the most preferred device was the Empatica E4 (SQI: 86%) [52]. Karoly et al. reported that seizures are phase-locked, preferring to occur during specific HR cycles by tracking seizure cycles using mobile seizure diaries and smartwatches; 10 out of 19 participants had seizures phase-locked to multiday HR cycles [59].

Many devices on the market have design deficiencies that pose limitations and affect overall patient acceptance and adherence. Occasional refusal of usage and negative responses were noted, particularly in the 60- to 69-year-old age groups (p < 0.05) [52]. The unpredictability of seizures poses a challenge in the seizure prediction field [52,60]. Another concern is the quality of the data recorded [61]. Wrist and ankle-worn devices showed a high sensitivity of 91% in identifying tonic-clonic seizures (TCSs); in contrast, high false alarm rates (FARs) were detected, mainly in wrist-worn devices (2.5/24 hours). Studies failed to mention the number of focal seizures experienced during the encounter of TCS in addition to whether they were tonic, clonic, included focal, or bilateral TCS [57]. HR and sleep parameters can be seizure drivers, measured from smartwatch devices. However, they are not helpful substitutes for polysomnography and electrocardiography as they are subject to errors and artifacts [62]. The wearable electrocardiography device to measure HR variability and detect seizures was feasible in patients with ictal autonomic changes. Before the value of 120 heartbeats exceeds the cutoff, the device considers it a positive seizure detection. It is important to note that not all seizures have ictal HR or autonomic changes [63]. Currently, the market lacks wearable devices that detect seizures other than TCS [64], and devices under study failed to detect all types of seizures [65,66].

Parkinson’s Disease (PD)

Patients with PD face the risk of falls and balance loss due to common symptoms such as bradykinesia, posture, and rigidity [67,68]. However, the advent of new technology devices that detect movement with the assistance of installed video cameras and motion and sound sensors brings hope for the early detection of these symptoms [69]. These devices, which can be used as an alternative to the scale scores used in PD, have been proven effective by literature [70]. The most common wearable devices used are smartwatches and beds (60%) to monitor motor impairments in neurodegenerative diseases, with smartwatches being particularly effective at monitoring seizures and tremors [67]. Although they can be used to detect early-stage symptoms of PD, only a few devices are FDA-approved, raising privacy and security concerns [67,71].

Bradykinesia is a classic symptom of PD; it can be monitored in multiple ways, such as gyroscopes, accelerometers, and magnetic, touch, and electromagnetic sensors [71-73]. Neurologists’ results were proportional to those obtained from accelerometers and gyroscopes, demonstrating its effectiveness; most motor symptoms assessed showed strong criterion validity with standard scales (R > 0.65). Moreover, when compared to the Dot Slide Test, measures of bradykinesia had high specificity and sensitivity of 88% and 95%, respectively [70].

Another common symptom of PD is tremors, which occur in the early stage of PD [74]. The sensor systems within the devices can identify the type of tremor and collect related data [75-77]; these systems can also monitor PD long-term and assess the severity of the disease. A study found that essential tremors can be differentiated from early-stage PD by machine learning (ML) using several parameters, such as the Arm-Symbolic Symmetry Index, which was found higher in PD by 16.0%, while the 180° Turn-Max Sagittal Angular Velocity; standing from a chair was smaller in PD [78].

The prognosis of gait in PD is mainly assessed by gyroscopes and accelerometers, which provide kinematic and dynamic parameters [71,79,80]. The risk of falls has been studied in patients with PD using wearable devices such as McRoberts and DynaPort Hybrid System light-weight sensors, which were able to assess balance and provide continuous feedback; patients with impaired quality of turning while walking had significantly higher jerkiness (p < 0.05) than ones not facing difficulty [81]. The opal inertial sensor, worn on the lumbar spine to track walking and turning in PD patients, revealed that PD patients took a higher number of steps to turn and exhibited an average of 67.3 turns/h compared to controls 65.2 turns/h; this allowed patients to take the appropriate measures to prevent and eliminate the risk of falls [82,83].

The quantification of muscle rigidity has been proposed in several studies; one study used a magneto-inertial motion tracking system, which targets the affected body parts and sets sensors to report the joint movement rigidity [84]. Sensors that document the movement instability regularly during activities could also be used by placing them on the patient’s back; an example is the BioKin device worn on the shoulders and iliac spine [85]. Wearable devices in PD mainly cannot be used in off-stage states, in the elderly, or in severe cognitive or motor impairments, which is considered a limitation [73,86].

Neurologic Rehabilitation

Ataxic gait and neurodegenerative ataxias are increasingly monitored by wearable technologies [87]. Cerebellar direct current stimulation is a proven effective method that acts on synaptic plasticity. Recently, transcranial alternating current stimulation was introduced as a therapeutic intervention to modulate cerebellar excitability. During the randomized, double-blind phase, the marginal mean difference was +11.0 points (95% CI: +9.3 to +12.7, p < 0.001) in the International Cooperative Ataxia Rating Scale and +4.1 points in the Scale for the Assessment and Rating of Ataxia (95% CI: + 3.5 to + 4.7, p < 0.001) suggesting an improvement in the real treatment group compared to the placebo group [88]. According to a clinical trial, robot-assisted gait training is an effective tool for gait training in patients with cerebellar ataxia. The improvement rate for 10-minute walking time was 19.0% and 29.0% for six-minute walking distance compared to therapy-assisted training [89].

Following a stroke, daily activities are impaired due to a long-term reduction in the functionality of the upper extremities [90]. The most common device used in outpatient settings is wrist accelerometry, which measures the activity of the upper extremities [91]. Wearable sensor data are used to provide performance feedback during home-based upper extremity training, which supports the assessment of impairment and areas related to recovery [92]. Schwerz de Lucena et al. found promising results: Over time, the patient’s hand use intensity increased (p = 0.012, slope = 9.0 hand counts/hour per day) using the manometer device compared to the control group (p-value = 0.059; slope = 4.87 hand counts/hour per day) [93]. Myoelectric interface for neurorehabilitation training is another wearable device proven to be feasible and tolerable for home-based training in stroke survivors chronically and severely impaired [94]. During rehabilitation, robot-assisted gait training is implemented to enhance the ability to walk [95]. Stationary rehabilitation robots such as ReWalk are also used [96,97]. Recently, wearable rehabilitation robots have been introduced, which are smaller in size and lighter, serving as an advantage in daily life training; improvements in spasticity and balance have been reported (random effects model, mean difference = 4.64, 95% CI = 3.22-6.06, p < 0.01) in Berg balance scale score [98,99]. However, the results for the usage and efficacy of wearable devices to improve walking activity following stroke remain mixed [93].

Multiple sclerosis patients require frequent monitoring and measurements of their condition [100,101]. Wearable sensor technologies detect subtle physical activity using multiaxial accelerometers, gyroscopes, and, recently, phone-based monitoring. Promising areas that can also be assessed using wearable devices in patients with multiple sclerosis are tremors [102], gait [103-105], postural control, and balance [106].

Implementing online connectivity with wearable devices will help greatly by providing constant feedback to subjects, which will positively reflect on compliance [52]. Wrist and ankle-worn devices showed high sensitivity in identifying TCSs. In contrast, high FARs were detected, mainly in wrist-worn devices. Future studies should focus on attempting to reduce FAR [57].

Literature reported that neurologists are willing to integrate artificial intelligence (AI)-assisted remote patient monitoring solutions, including non-wearable and wearable sensor attachments and smartwatches, in managing PD by early detection of the advanced stages using AI [107,108]. Tattoos and fabrics can be implemented as noninvasive alternatives to wearable devices for neurodegenerative diseases to monitor daily impairment [67].

Role of wearable technologies in endocrinology

Thyroid Dysfunction

Early detection and continuous monitoring of thyroid dysfunction are essential for effective management and treatment. Wearable devices are increasingly utilized to monitor thyroid function, providing continuous data that can predict thyroid dysfunction. A study developed an ML-assisted system that used HR data from wearable devices to predict thyrotoxicosis in patients. HR data were collected over four months from 175 patients, with three to four thyroid function tests performed monthly. The system achieved a sensitivity of 86.14% and a specificity of 85.92%, highlighting its potential for clinical application. The PPV was 52.41%, and the NPV was 97.18%. When excluding subclinical thyrotoxicosis, the sensitivity remained at 86.14%, but specificity improved to 98.28%, with a PPV of 94.57% and an NPV of 95.32%. This study demonstrates that the HR parameters obtained from wearables can be an objective indicator for monitoring thyroid function, potentially reducing the need for frequent blood tests [109].

Another study highlighted the advantages of continuous monitoring systems in thyroid dysfunction management. Continuous monitoring of thyroid function using digital tools provides real-time feedback to patients and healthcare providers. In this study, HR changes measured by wearables were compared to T4 changes, demonstrating that HR could predict changes in T4 levels with a correlation coefficient of 0.75. The study found a strong correlation between resting HR and T4 levels, indicating that wearable devices can effectively monitor thyroid function over time. This continuous monitoring can alert healthcare providers to significant changes in a patient’s condition, enabling timely interventions and adjustments to treatment plans, thus improving overall patient outcomes [110].

Fertility Tracking

Wearable devices have revolutionized the tracking of the fertile window in women, defined as the six days preceding ovulation. Various devices and wearables are marketed for this purpose, using biomarkers such as basal body temperature (BBT), urinary hormone levels, and other physiological parameters. The Ava bracelet incorporates parameters beyond BBT, including resting HR, HR variability, and respiratory rate. It accurately detected a six-day fertile window in 95% of cycles, with temperature and respiratory rate lower in the fertile window and HR higher in the early and late luteal phases. However, a challenge women face with the Ava bracelet is its dependency on the user's adherence to wearing it nightly and syncing the data, which can lead to gaps in data collection and potential inaccuracies.

In comparison, the FemSense ovulation detection patch detected ovulation in 81% of cycles, with a BBT rise within one day of ovulation in 57% of cycles. The patch is single-use and requires users to purchase multiple patches over time, adding to the overall cost. The Natural Cycles application uses an algorithm to predict ovulation, showing a mean delay of 1.9 days between the first positive urinary luteinizing hormone (LH) and the estimated ovulation day. Despite its accuracy, the app relies heavily on user input, which can lead to errors if the data is not consistently or accurately recorded. Another wearable, the Oura ring, measures nocturnal temperature and HR, which were significantly lower on the day of and the day after a positive urinary LH and higher during the mid- and late-luteal phases. These devices offer different advantages, with the Ava bracelet providing a comprehensive set of physiological parameters, while the FemSense patch and Natural Cycles app offer ease of use and specific biomarker tracking [111].

Metabolic Health and Weight Management

Digital technologies have been extensively used for weight management. A systematic review found that digital tools, including mobile apps and web-based platforms, effectively support weight loss in overweight or obese individuals. These technologies facilitate self-monitoring, goal setting, and personalized feedback, reducing weight significantly. For example, one study reviewed found that users of digital weight loss interventions lost an average of 2.24 kg more than those in control groups without such interventions over six months [112].

A nine-month RCT evaluated a web- and app-based intervention to promote a healthy lifestyle and weight loss among social welfare and healthcare employees. The intervention utilized the “Healthy Weight” web platform and the “MyFitnessPal” mobile app to assist participants in tracking their dietary intake and physical activity. Participants were provided with personalized feedback, dietary recommendations, and exercise plans. The mobile app enabled real-time tracking and goal setting, ensuring users stayed engaged with their health goals throughout the intervention period. The intervention led to a significant average weight loss of 5.3 kg (11.7 pounds) and improvements in lifestyle behaviors, demonstrating the effectiveness of digital interventions in workplace settings. Participants in the intervention group showed a 15% increase in physical activity levels and a 20% improvement in dietary habits compared to the control group. These results underscore the potential of digital tools to enhance health outcomes in various settings. However, challenges were noted in the implementation of these digital interventions. Ensuring high adherence and consistent data collection was difficult, with some participants struggling to maintain engagement over the nine months.

Additionally, the reliance on user self-reporting for dietary intake and physical activity posed a risk of inaccurate data entry. Technological issues, such as app malfunctions and difficulty syncing data between devices, also affected the overall user experience. These challenges highlight the need for ongoing support and improvements in digital intervention designs to maximize their effectiveness and user adherence [113].

Another study by Burke et al. evaluated the AKTIDIET® mobile app, designed to reinforce health advice and promote weight loss. Despite the initial success in weight reduction, with participants in the intervention group losing an average of 4.5 kg over 12 weeks compared to 2.4 kg in the control group, the study found no significant differences in blood cholesterol levels (low-density lipoprotein: -0.05 mmol/L vs. -0.03 mmol/L), BP (systolic: -2.1 mm Hg vs. -1.8 mm Hg; diastolic: -1.3 mm Hg vs. -1.0 mm Hg), or adherence to dietary and physical activity recommendations between the intervention and control groups. This shows that high-quality design and patient involvement in app development are crucial to enhancing usability and efficacy [114]. On the other hand, a study was conducted to test the effect of feedback specifically. The SMARTER randomized clinical trial evaluated the efficacy of tailored daily feedback (FB) and lifestyle self-monitoring on weight loss. The study involved 502 participants with a BMI between 27 and 43 kg/m², who were divided into two groups: one receiving self-monitoring plus feedback (SM+FB) and the other only self-monitoring (SM). Participants used a Fitbit Charge 2 for physical activity tracking and a digital scale for daily weighing. The SM+FB group received up to three daily personalized feedback messages via a custom smartphone app. After six months, both groups showed significant weight loss (SM+FB: -3.16%, SM: -3.20%), but there was no significant difference between the two groups (p = 0.940). Despite the lack of a significant difference between groups, the results demonstrated the potential of digital interventions for weight management, particularly in providing scalable and accessible support for individuals with obesity. This study further proves the importance of self-monitoring in weight loss. It suggests that the additional feedback provided by the app did not significantly enhance outcomes beyond self-monitoring alone. This finding highlights the potential need for more personalized or interactive forms of support to achieve more significant weight loss [115]. Several challenges and limitations were shared in these studies, such as user engagement and adherence, which were critical factors influencing the effectiveness of these tools. Continuous engagement strategies and personalized feedback are essential to maintain user motivation and achieve sustainable weight loss [113-115].

Another RCT was done to evaluate the effectiveness of a smartphone-based weight management app called Noom on metabolic parameters in adults. The study involved 129 participants randomly assigned to three groups: control, the app only (diet and exercise self-logging), and an app with personalized coaching from professional dieticians and exercise coordinators. Assessments were conducted at baseline, week 6, week 12, and week 24. Results showed that the app with personalized coaching group experienced more significant body weight reductions (control: -0.12 kg; app only: -0.35 kg; app with personalized coaching: -0.96 kg) and body fat mass reductions (control: -0.13 kg; app only: −0.64 kg; app with personalized coaching: -0.79 kg). These results highlight the potential benefits of personalized coaching in digital health interventions for weight management [116]. Improving personalization and in-app engagement is vital to overcoming adherence issues that most users face [113-116].

Diabetes

Wearable devices have become crucial in managing diabetes, offering real-time monitoring and feedback to patients and healthcare providers. These devices include CGMs, smartwatches, and fitness trackers, providing data on blood glucose levels, physical activity, and other vital parameters.

Studies have shown significant improvements in glycemic control and patient adherence with these technologies. For instance, the FreeStyle Libre and Dexcom G6 are CGMs that measure glucose levels in interstitial fluid through a small sensor inserted just under the skin. These devices provide real-time glucose readings and trends without fingerstick calibration. The FreeStyle Libre has a mean absolute relative difference (MARD) of 9.2%, and the Dexcom G6 has a MARD of 9.0%, indicating high glucose reading accuracy. User satisfaction with these devices is high due to the convenience of CGM without needing needle sticks [117-119].

In a randomized clinical trial involving 27 participants, integrating metabolic expenditure information from wearable fitness sensors into an AI-augmented automated insulin delivery system significantly improved glycemic control in patients with type 1 diabetes. The metabolic information included physical activity levels, HR, and caloric expenditure data. During the 76-hour study, the AI-augmented system maintained better glucose control with clinical time in range targets of 71.2% and 75.5% and time below the 1.0% and 1.3% range, respectively [120]. Moreover, a noninvasive glucose monitoring system based on single-wavelength PPG was evaluated. This technology could accurately track glucose levels without invasive procedures, with a MARD of 7.62%. In addition, more than 95% of the predicted glucose values were within the clinically acceptable zone, offering a convenient alternative for patients [121]. Similarly, a study by Bartolome et al. evaluated the iGLU 2.0 wearable device, an accurate, noninvasive continuous serum glucose measurement within an internet of medical things framework. It allows for real-time remote monitoring capabilities by healthcare providers, enabling monitoring and early intervention by healthcare providers [122]. Results showed that the iGLU 2.0 device had a significantly lower MARD of 4.86% in measuring serum glucose levels compared to single-wavelength PPG (7.62%), demonstrating a higher accuracy [121,122]. The device has shown superior linearity (97%) and a high measurement range (80-420 mg/dl) compared to other noninvasive [122].

Wearable devices are also improving the detection of hypoglycemia. A study using smartwatch data demonstrated the feasibility of noninvasive hypoglycemia detection by monitoring physiological parameters such as HR and skin conductance (electrodermal activity); this allows for early detection of hypoglycemia, thus encouraging early interventions by alerting users and caregivers [123]. Moreover, wristband data, including step count, HR, and caloric expenditure, has been used to determine physical activity characteristics. This data aids in the effectiveness of automated insulin delivery systems by providing real-time feedback on the user's physical activity levels. Thus, adjusting insulin delivery based on physical activity allows for maintaining optimal blood glucose levels and preventing hypoglycemia during exercise, thereby improving overall glycemic control [124].

Role of wearable technologies in orthopedics

The role of wearables in orthopedic rehabilitation has become more crucial due to the difficulties in adhering to traditional methods such as exercise and drug therapy. The lack of adherence poses increased risks of significant complications varying from fractures to disability and osteoarthritis. In-person physical therapy, although effective and widely used, requires increased adherence and close supervision by medical staff. Wearable devices may improve patients’ adherence and outcomes by allowing customized, precise, and targeted rehabilitation programs while relieving the burden of direct and close supervision by medical staff, thus making them superior to alternative therapies [125].

Gait analysis provides vast information on an individual’s gait, which has a variety of uses, such as early detection of abnormal gaits or in treatment and monitoring the progress of physical rehabilitation. This is done through intelligent systems that incorporate vision and locomotion sensors. However, there is concern regarding the accuracy of the data, which can be affected by occlusion to sensors, viewing angles, and clothing patterns, which must be considered when analyzing the data [126]. Wang and Yan proposed a more advanced gait recognition that considers different view angles with the help of a novel algorithm that combines several gait learners to provide more accurate data [127]. Thus, this information may be applied in physical rehabilitation to personalize the individual's therapy, allowing for better outcomes in less time [126]. Another study showed that wearables can be used in continuous monitoring following elective hip, knee, and spine procedures to predict recovery outcomes [128].

Wearable devices used in rehabilitation focus on motion trackers that provide precise data, which is, in turn, interpreted by a system that allows for close and accurate monitoring while providing immediate feedback. This remote monitoring system enables physical therapy from home, with the same medical attention and accuracy as in-person physical therapy. A study by Correia et al. showed that patients engaged in digital remote rehabilitation have improved outcomes compared to in-clinic rehabilitation, specifically in performance tests, range of motion, and patient-reported outcomes [129]. Another study by Mehta et al. used a wearable activity monitor in patients following hip and knee arthroplasty to remotely assess and aid patients post-operatively. The study, which involved 242 patients (118 intervention, 124 control), used a wearable activity device that tracks steps, sends reminders to patients regarding milestones postoperatively, assesses pain scores, and connects the patient to a clinician upon need. The study showed a significantly reduced rehospitalization rate in the intervention group (3.4%) compared to patients who were not monitored remotely (12.2%). This allows for postoperative guidance, improved engagement and adherence, and early medical intervention, preventing complications [130].

Additionally, to further demonstrate telerehabilitation’s efficacy, a clinical trial aimed at determining the effectiveness of telerehabilitation in stroke patients has shown improved results in the intervention group compared to in-clinic physical therapy. The intervention group showed higher adherence (98.3%) to physical therapy sessions compared to the in-clinic physical therapy (93.3%) [131]. Moreover, telerehabilitation was just as effective as conventional therapy following total knee arthroplasty [132]. The effectiveness of wearables and smart devices in patient outcomes significantly reduces the burden of conventional in-person, close-monitoring rehabilitation on medical staff. It significantly addresses the issue of compliance [129-132].

Other noteworthy uses of wearables in orthopedics include a novel neck brace that provides adjustable neck support with varying stiffness while avoiding strain on antagonist muscles in patients with dropped head syndrome. This passive device provides head support with minimal muscle effort during motion in the sagittal plane. However, the device was only tested on non-effected individuals yet showed promising results with minimal discomfort and increased range of motion, thus surpassing all previous models. Nonetheless, further investigations and trials are required to validate the system’s safety for broad clinical use [133].

The widespread back and neck pain due to poor posture has led to the development of wearable devices that can monitor posture and provide real-time feedback for correction. The device contains sensors that can monitor sitting, standing, walking, and lying down positions with a sensitivity of 95.84%. This increases the patient's awareness of their posture, allowing them to improve it and eventually reduce the burden of chronic pain [134]. Another study included 26 physiotherapy patients complaining of nonspecific low back pain with medium and high-risk chronicity; the control group was asked to continue physiotherapy as usual, while the 12 participants in the intervention group were given a wearable-based walking intervention in addition to physiotherapy. Over 26 weeks, the experimental group showed a significantly lower pain score of 1 and 3.36 in the control group.

Additionally, the intervention group had a lower disability score (12.64) than the control group (19.45). This study, though it didn't include many participants, verified the effectiveness of wearables in reducing chronic pain. It showed the value of wearable devices in increasing adherence to physical activity, which, in turn, has been shown to improve patient outcomes [135].

Role of wearable technologies in cancer

Wearable devices have been receiving attention in the field of cancer. Yet, a major downside was adherence and acceptance of these devices since the patients are already under the burden of the disease and its side effects [136]. Wearable devices have facilitated physical activity in patients with cancer by creating a form of self-awareness with the help of the activity tracker [137]. Literature reported that physical activity can enhance the QOL and prolong survival [138,139]. Fitness can minimize cancer-related side effects in breast cancer survivors [140]. While colorectal cancer survivors benefited from telephone-delivered health coaching [141], Lynch et al. used the Garmin Vivofit 2 wrist-worn device for breast cancer survivors, which displayed inactivity alerts, calories, steps, distance, rest, and sleep time. Coaching was combined with the mHealth devices, which successfully increased physical activity (69 min/wk; 95% CI = 22-116; p < 0.01) and reduced prolonged (-42 min/d; 95% CI: -83 to -2; p = 0.04) and total sitting time (-37 min/d; 95% CI: -72 to -2; p = 0.01) [142]. Trinh et al. revealed that sedentary behavior was significantly reduced (455.4 weekly minutes), and moderate-to-vigorous physical activity increased to (+44.1 weekly minutes) using a combination of jawbone wearable devices and web-based intervention [143]. Alberts et al. used Spire Health tag and Spire Stone clip-on devices for respiratory and chronic pain monitoring in adult patients who survived childhood cancer; the results supported these devices' acceptability (85.7%) and feasibility [144]. Hardcastle et al. demonstrated that physical activity was improved (49.8 min/week (95%CI): 13.6-86.1, p = 0.007) using distance-based health coaching and wearable technology compared to standard practice [145]. Patients in cancer rehabilitation centers provided positive perceptions and willingness to use wearable exercise trackers daily in both inpatient (58%) and outpatient (76%) settings to increase physical activity [146]. A barrier to these devices is the inaccurate feedback and technological problems faced and described in several studies [144,147-149].

Liu et al. aimed to predict death within a seven-day window using wearable devices and AI in patients with terminal cancer receiving end-of-life care, which occurred in various settings using smartwatches to monitor patient progression, and the data of the device were successful in clinical predictions (XG Boost model F1-score 78.5%, 93% accuracy, and 97% specificity), with average HR being the most important factor [150]. The results were consistent with other studies [151,152].

Remote early warning scoring (REWS) is a system that uses a wireless wearable accelerometer patch (HealthDot) and measurements to detect deterioration following major abdominal cancer surgeries (REWS sensitivity: 0.20 95% CI: (0.13-0.29), specificity: 0.96 95% CI: (0.95-0.97)). The results were comparable to the modified early warning score (MEWS) (MEWS sensitivity: 0.20 95% CI: (0.13-0.29), specificity: 0.96 95% CI: (0.95-0.97). It can serve as a clinical decision support tool [153].

Wearable technologies and physical activity trackers have been proven effective in improving health outcomes, and future cancer research should focus on integrating them for long-term use. The field of cancer detection using wearable sensor systems also requires the incorporation of AI in future research [154].

Role of wearable technologies in mental health

Wearable technology has significantly impacted healthcare by providing innovative tools for continuous monitoring. These gadgets, which range from sophisticated biosensors to smartwatches, offer real-time data on vital signs related to mental health, such as stress levels, physical activity, and sleep patterns. They contribute to improved diagnosis, management, and patient outcomes, while recent advancements and future directions continue to shape their evolving role in mental health care.

Yen conducted a study to assess the impact of smart wearable devices on healthy behavior and QOL of healthy adults. The findings revealed that participants using smart bracelets or smartwatches demonstrated positive changes in several aspects of a healthy lifestyle and QOL, especially for those with more advanced features and capabilities. The outcomes showed that wearable devices could be helpful in early psychological interventions to target changes in health-related behaviors. However, issues like maintaining the user engaged in the long run and understanding the basic vs. advanced features were observed [155].

In addition, Lee et al. assessed the efficacy of MAVE®, a neurofeedback wearable device, in stress alleviation via meditation. Participants using neurofeedback-assisted meditation showed significant reductions in perceived stress and higher device satisfaction compared to those with non-assisted meditation. However, some issues were observed, such as mild and temporary adverse effects like headaches and possibly inaccurate interpretation of the electroencephalogram (EEG) signals. The outcomes showed that neurofeedback-assisted wearables can help manage stress and improve psychological well-being [156]. Furthermore, Zalta et al. examined the efficacy of the Re-timer®, a wearable light device on the arm, in decreasing post-traumatic stress disorder (PTSD) and depression symptoms. The active Re-timer® device produced clinically significant improvements in sleep quality and decreases in PTSD and depression symptoms in study participants, with effect sizes of 0.94 and 0.74, respectively. The findings confirmed that wearable light therapy for PTSD is possible, feasible, and well-accepted by the participants; however, issues like compliance with the therapy and side effects like mild headaches were identified. These results suggest that wearable light devices may be useful as additional therapies for PTSD in future research [157].

In addition, Nuss et al. examined the synergistic impact of wearable fitness trackers (WFT) and motivational interviewing (MI) on physical activity and motivation. WFTs worn on the wrist in combination with MI led to increased autonomous motivation and basic psychological need satisfaction, but there was no change in physical activity levels. The study showed the positive impact of combining WFTs with MI for motivation: participants with a high level of compliance used wearable devices. However, challenges observed in the study were that although there was increased motivation, there were no significant changes in physical activity [158]. Moreover, Liau et al. (2018) examined how the mental contrasting with implementation intentions (MCII), self-regulation strategies, can promote physical activity and enhance mental health. The participants wore or clipped Fitbit Zip trackers to measure daily steps and participate in MCII activities. The outcomes revealed increased physical activity levels and enhanced mental well-being, especially among women. Notably, the researchers noted high compliance with the use of the Fitbit devices. The devices provided accurate tracking and improved motivational goals. These outcomes suggest that WFT devices with self-regulation approaches may be useful in increasing physical activity and improving mental health [159].

In addition, Smith et al. examined the effectiveness of a stress-management intervention that uses wearable technology on employees’ mental health. Employees of a large technology corporation were recruited in this study. They wore wearable devices that measured respiratory patterns and offered biofeedback on a smartphone application for four weeks. The treatment group reported 15.8% fewer negative instances of stress, a 13.0% reduction in stressful signs, and a 28.2% decrease in the days the experimental group felt anxious or stressed compared to the control group. Still, there was not enough evidence showing that general health checkups had enhanced the broad measures of well-being. The limitations were difficulty translating intervention content to the different contexts of workplaces and ensuring device utilization and compliance with the intervention [160].

Similarly, Arsalan and Majid investigated a framework to measure trait anxiety using the Muse EEG headband’s resting state EEG data. This study showed that the random forest (RF) classifier yields the highest accuracy at 87.69% for two-class and 83.07% for three-class anxiety classification. The real-time data offered by the wearable EEG device was necessary for early-stage detection and monitoring of anxiety because it allowed continuous, noninvasive measurement of brain activities. Some of the issues the researchers identified were related to data quality, dealing with noises when the experiments were conducted in uncontrolled environments, and validating the findings across different populations. The results suggest that wearable EEG technology, such as the Muse headband, has a high potential for the future of personalized healthcare and constant screening of mental well-being [161].

Further, Tazawa et al. also investigated the feasibility of wristband-type wearable devices integrated with ML to detect depression and its severity level. The results showed significant differences in HR, steps, and sleep duration between depressed patients and healthy subjects. The ML model achieved 0.76 accuracy in identifying symptomatic patients. Nonetheless, the challenges were continuous and accurate data collection and concerns about reliability when dealing with wearable metrics. The results indicate that wearable devices could be promising tools for the objective assessment and early diagnosis of depression, which might lead to improvements in the management of depression [162].

Pedrelli et al. used wristband and smartphone sensors to monitor 31 patients with major depressive disorder (MDD) for eight weeks. They identified high compliance and significant correlations between the data gathered by sensors and clinician-rated measures of depression severity. There was evidence for the practicability of employing wearable devices for constant monitoring. However, several limitations were discovered, such as limited participants and technical problems impacting compliance. The outcomes provided evidence for applying digital phenotyping to continuously monitor and manage MDD and other psychological conditions [163].

Furthermore, Swanson et al. examined the feasibility of home-wearable light therapy for postpartum depression among eight participants. Depressive symptoms were reduced, though objective changes in circadian rhythms were minimal. Notably, the limitations included a small number of participants and the use of subjective data concerning adherence. The outcomes showed the effectiveness and the possibilities of using light therapy to treat postpartum depression [164]. Also, Muhammad and Al-Ahmadi designed an anxiety detection framework based on EEG data obtained from the Emotiv excess post-exercise oxygen consumption headset during exposure therapeutic sessions. Classification accuracy was high in the study; RF was the most accurate, with 94.90% and 92.74% for two- and four-level anxiety, respectively. It is essential to mention that wearable EEGs allow the monitoring of patients' conditions in real-time and timely intervention. However, there were two significant issues, such as the quality of data and user compliance with the intervention. The results showed the feasibility of using wearable EEG devices for anxiety disorder management [165].

Discussion

Wearable health devices stand at the forefront of modern chronic disease management, offering continuous monitoring and a personalized approach to patient care. This review aims to meticulously evaluate their effectiveness and the challenges they present in the context of cardiology, respiratory, neurology, endocrinology, orthopedics, oncology, and mental health. By dissecting their impact on diagnosis, prognosis, and patient outcomes, we seek to illuminate the significance of wearable technology in advancing healthcare across multiple medical disciplines.

Wearable devices have become increasingly prominent in cardiac monitoring, including disease detection for cardiac conditions such as AF and cardiac arrhythmia. These advanced devices, such as single-lead ECG patches, smartwatches, and custom wearable ECG recorders, provide continuous, real-time monitoring of the user's cardiac activity. This permits immediate detection and effective management of a variety of cardiovascular disorders. These devices make it easier to rapidly alert users or healthcare providers about irregular heartbeats, like AF, for early detection. It can be used to better understand how frequently and for how long arrhythmias occur, aiding in diagnosis and planning treatment. In addition, remote monitoring features enable physicians to access real-time data, allowing faster interventions or modifications to the treatment strategy. The potential impact of these devices on patient outcomes is significant, as they can lead to early detection and intervention, thereby reducing the risk of serious cardiac events and improving overall patient health [166,167].

Besides, wearable technology plays a significant role in diagnosing and managing respiratory conditions such as COPD and asthma by enabling continuous tracking of vital respiratory parameters like respiratory rate, oxygen saturation (SpO2), and minute ventilation, which helps identify exacerbations or deteriorations in respiratory function. Devices that measure oxygen saturation provide critical information, as low levels can indicate respiratory distress, prompting timely medical intervention. Additionally, wearable activity trackers monitor physical activity levels, which is essential for understanding the impact of respiratory conditions on daily life, where reduced activity may signal worsening symptoms or disease progression [168]. Advanced wearables can also analyze breathing patterns to detect irregularities, such as changes in frequency or depth, that may indicate an asthma attack or COPD exacerbation. Some devices assess air quality and environmental factors, like pollutants and allergens, which can trigger asthma attacks or worsen COPD symptoms, helping patients make informed decisions about outdoor activities. Furthermore, wearables facilitate remote patient monitoring, allowing healthcare providers to track patients’ respiratory health without requiring in-person visits, which is particularly beneficial for managing chronic conditions [169]. They can integrate data with smartphone apps and cloud platforms, providing actionable insights to adjust treatment plans based on real-time health information. By analyzing trends in collected data, wearables can predict exacerbations or acute episodes, enabling preemptive action and better disease management. Overall, wearables enhance the diagnosis and management of respiratory conditions by providing continuous, real-time data on vital signs, activity levels, and environmental factors, supporting personalized care, improving patient engagement, and leading to better health outcomes for individuals with COPD and asthma [170]. However, the effectiveness of the wearable devices in estimating the respiratory rate and oxygen saturation of the wearer was tested using a head-mounted device to provide breath-by-breath monitoring during cycling exercise. This device employed a formula to predict respiratory rate from changes in nostril pressure. It was tested on ten subjects using a variety of tests that measured the device’s performance compared to a reference device. The validity analysis with Bland-Altman, linear regression coefficient (r²), and percentage error (%E) was proven to be effective and precise, with a 4.03% overall %E for breath-by-breath data and 2.38% for 30-second averages. The bias of the breath-by-breath analysis was higher compared to the bias of the 30-second window analysis, ± 6.27 breaths/min and ± 1.60 breaths/min, respectively. Specific conditions were modeled, where the percentage error increase was 6.65%. Through these results, it is affirmed that the accuracy and reliability of the device are very high for respiratory rates during exercises, which makes this device useful for several indoor activities requiring respiratory rate monitoring without invasive devices [171].

On the other hand, wearable devices have demonstrated their application in detecting neurological disorders such as epilepsy and psychogenic nonepileptic seizures (PNES). These devices use accelerometer sensors to monitor motor activity, a critical seizure indicator. They enable continuous and unobtrusive monitoring of patients in natural settings, which is essential for capturing seizures outside of hospital environments. Using time-frequency analysis and advanced classification algorithms, these devices can effectively distinguish between ES and PNES. This distinction is vital because PNES are often misdiagnosed as ES, leading to inappropriate treatment. The wearable devices offer a high sensitivity in seizure detection and a low rate of false alarms, making them a reliable tool for early diagnosis and timely intervention in neurological disorders [172]. The wrist-worn accelerometer, in particular, demonstrates a high sensitivity of 100% and a specificity of 85.77% in detecting convulsive seizures. It effectively identifies seizure events while minimizing false alarms, ensuring reliable neurological monitoring. Proper device placement is crucial for accurate data collection, as incorrect positioning can lead to false positives. The continuous, unobtrusive monitoring provided by wrist-worn accelerometers is particularly beneficial for patients with nocturnal seizures, who face higher risks of injury or sudden death due to unnoticed seizures. Monitoring and analyzing motor activity through these devices aids in reducing diagnostic delays and optimizing treatment plans for patients with neurological conditions [173].

Additionally, wearable devices have shown significant contributions to managing endocrine disorders, particularly diabetes. These advancements include wearable artificial pancreas systems that integrate CGM with insulin pumps to automate blood glucose control. This integration results in superior glycemic control and reduces hypoglycemic events [174]. Additionally, smart insulin pens enhance diabetes care through dose tracking and real-time data sharing, improving accuracy and adherence. Furthermore, microneedle patches offer a minimally invasive method for transdermal drug delivery, effectively administering hormones and therapeutic peptides without the pain and inconvenience of injections. Implantable devices provide long-term, controlled release of medications, significantly improving adherence and glycemic control in patients with diabetes [175]. These technologies improve the pharmacokinetics and pharmacodynamics of endocrine therapies and enhance patient adherence and QOL. CGM systems are essential for managing diabetes, providing real-time glucose levels and trends data. These systems have demonstrated notable sensitivity and specificity, making them reliable for clinical use. The sensitivity of a CGM system refers to its ability to correctly identify actual glucose level changes, while specificity indicates its capacity to avoid false detections of such changes. Several studies have evaluated the accuracy of different CGM systems [117]. For instance, the Medtronic Guardian system has a MARD of 15.8%, the Dexcom Seven Plus has a MARD of 16.7%, and the Abbott Navigator shows a MARD of 12.8%. These values reflect the percentage difference between the CGM readings and reference blood glucose values, with lower MARD values indicating higher accuracy. Over the past decade, advancements in CGM technology have significantly improved the accuracy of these devices, making them safe for therapeutic decisions such as insulin dosing. For example, the Enlite sensor by Medtronic, launched in 2011, offers enhanced accuracy and user comfort compared to earlier models. These improvements have been crucial in reducing the rate of false alarms and enhancing the reliability of glucose monitoring, which is essential for effective diabetes management and patient safety [176].

Similarly, wearable devices are increasingly significant in managing musculoskeletal conditions and preventing injuries. These devices use advanced sensors to monitor musculoskeletal health, providing critical data for injury prevention and rehabilitation. Equipped with nanogenerators and sensors, wearable devices track joint movements and muscle activity in real time, helping detect abnormal patterns indicative of musculoskeletal issues and allowing for early intervention. This feedback on joint angles and muscle contractions is essential for athletes and individuals undergoing rehabilitation to ensure proper form and avoid further injury [177]. Wearable sensors also analyze gait patterns to identify irregularities that might lead to injuries such as plantar fasciitis, shin splints, and other lower extremity problems, and they monitor the progress of patients recovering from surgeries to ensure proper walking mechanics. In rehabilitation settings, these devices provide valuable data to tailor physical therapy programs to the specific needs of patients, tracking recovery progress by measuring range of motion, strength, and endurance. This data-driven approach ensures effective rehabilitation exercises and adjustments based on patient progress. Wearable devices can predict potential injuries by analyzing patterns and identifying risk factors such as muscle fatigue, improper technique, or overuse. This allows athletes to monitor their training load and receive alerts to prevent overtraining or injury. This proactive approach is crucial in sports, where preventing injuries can significantly enhance performance and longevity. Additionally, advancements in wearable technology, such as nanogenerators that harvest biomechanical energy from body movements to power the devices, address battery limitations, making long-term use more feasible [178]. Self-powered wearable devices are particularly beneficial in orthopedics, where continuous monitoring without frequent battery changes is essential for effectively managing musculoskeletal conditions. These technologies not only aid in managing and preventing musculoskeletal conditions but also support personalized medicine approaches, offering tailored interventions based on individual patient data [179]. The effectiveness of devices in detecting abnormal movement patterns and their associated sensitivity and specificity can be assessed based on the provided document. These devices, including ML models, wearable sensors, and video analyses, are used to classify agility movement tasks, predict peak knee abduction moments (KAMs), and assess the risk of anterior cruciate ligament injuries [180]. The classification models developed for detecting high or low KAMs showed an overall accuracy of about 80% and an area under the curve of 0.81-0.85. The best classification model, the support vector machine (SVM) Fine Gaussian model, demonstrated a true positive rate (TPR) of about 90% in testing, indicating high sensitivity in identifying trials generating high KAMs. PPVs were 74.7% for the mean kinematics model and 69.4% for the peak kinematics model. However, regression models for predicting peak KAM values from kinematic features showed suboptimal accuracy, with less than 50% of the variance explained (R² ranging from 0.33 to 0.46). The root mean square error for the best regression models ranged from 0.936 to 1.078 Nm/BW, which is high given the typical range of KAM values. In contrast, deep learning-enabled smart socks based on triboelectric nanogenerators for gait detection achieved an identification accuracy of 93.54% and an activity detection accuracy of 96.67%. The TPR of 90% for the SVM Fine Gaussian model in classifying high KAM tasks indicates high sensitivity, and the smart socks for gait detection also showed high accuracy, implying good sensitivity. While the document does not provide explicit specificity values, the high PPV for the classification models suggests reasonable specificity. The regression models, however, demonstrated lower performance and specificity in predicting exact peak KAM values [181]. Overall, the devices and models show promise in detecting abnormal movement patterns with good sensitivity, especially in classification tasks. However, the accuracy of regression models for predicting specific kinetic values is less optimal.

Furthermore, wearable devices’ transformative role in cancer care offers new paradigms in early detection, monitoring, and diagnosis. These devices, integrated with internet of things sensors, enable real-time monitoring of various health parameters, providing crucial information for patients and physicians. One of the most significant advantages of wearables is their potential for early cancer detection, as they can continuously monitor health indicators that may signal the onset of the disease, allowing for earlier intervention. In addition, these devices play a crucial role in diagnosing cancer by collecting and transmitting detailed health data in real time, which can be analyzed to identify patterns or anomalies that may suggest the presence of cancer [182]. They can also monitor the progression of the disease or the effectiveness of treatment, offering insights that can inform adjustments to the therapeutic approach. Another emerging application of wearables in cancer care is remote clinical trial monitoring, offering a cost-effective solution for collecting patient data outside of traditional clinical settings and providing valuable data that can help tailor treatments to individual needs and improve outcomes. The integration of wearable devices into cancer care represents a significant advancement in the early detection, monitoring, and diagnosis of the disease, with the potential to improve patient outcomes, personalize treatment plans, and revolutionize the approach to clinical trials. As technology continues to evolve, the role of wearables in cancer care is expected to expand further, offering new opportunities for improving patient care and treatment efficacy [183].

For instance, they have become valuable tools in monitoring indicators of mental health conditions such as stress and depression. These devices leverage advanced sensors to detect physiological signals associated with stress and depression, including HR variability, skin conductance, BP, and brain activity. For example, smartwatches and wristbands can measure HR and BP, while EEG headbands assess brain activity. Wearables like rings and patches monitor skin conductance to detect stress levels, and newer technologies, such as near-infrared spectroscopy, provide insights into brain responses to stress. These devices offer a noninvasive, user-friendly way to collect continuous data, enabling real-time monitoring and early detection of mental health issues. Furthermore, the integration of AI enhances the analysis of collected data, providing personalized feedback and interventions to manage stress and depression effectively [184].

## Conclusions

With their potential for real-time monitoring and enhancing patient outcomes, wearable health devices have shown considerable promise in managing chronic illnesses. They not only provide rapid and continuous access to crucial health data for patients and healthcare professionals but also significantly improve patient outcomes. This shift in managing chronic illnesses encourages people to become more involved in healthcare, which enhances treatment compliance and patient involvement. Additionally, they give medical professionals faster, more accurate information to help them make decisions. However, this increased dependence on wearable technology highlights essential issues that must be resolved, such as protecting patient privacy and guaranteeing data veracity. The data these devices collect must be accurate for them to be used effectively in therapeutic settings; any discrepancies might compromise their dependability and credibility. It is essential to overcome the obstacles posed by wearable health devices in order to fully realize their benefits in the management of chronic diseases. To overcome these obstacles and optimize wearables' advantages in treating chronic diseases, researchers, healthcare professionals, and technology developers must continue to innovate and collaborate. By developing and integrating these technologies, the healthcare industry may move closer to a future where chronic illnesses are managed more skillfully, with better patient outcomes and more economical use of healthcare resources.
